# A Novel Test of Dynamic Visual Function: Comparison Between Presbyopic and Non-Presbyopic Individuals

**DOI:** 10.3390/diagnostics16121914

**Published:** 2026-06-20

**Authors:** Bingqing Sun, Yuhao Ye, Xingtao Zhou, Ye Xu

**Affiliations:** 1 Eye Institute and Department of Ophthalmology, Eye & ENT Hospital, Fudan University, Shanghai 200031, China; 17301050234@fudan.edu.cn (B.S.);; 2 Key Laboratory of Myopia and Related Eye Diseases, National Health Commision (NHC), Chinese Academy of Medical Sciences, Shanghai 200031, China; 3 Shanghai Research Center of Ophthalmology and Optometry, Shanghai 200031, China; 4 Shanghai Engineering Research Center of Laser and Autostereoscopic 3D for Vision Care, Shanghai 200031, China

**Keywords:** presbyopia, additional diopter, sharpness discrimination, integration, stereopsis

## Abstract

**Background/Objectives:** Given the limited evidence on multi-distance visual function assessment in presbyopia, this study aimed to compare dynamic binocular visual function between presbyopic and non-presbyopic (NP) participants at different distances, and to further evaluate the effects of additional power (ADD) on dynamic sharpness discrimination, binocular integration, and dynamic stereopsis in presbyopic participants. **Methods:** A total of 54 presbyopic and 77 NP participants were tested at 0.4 m, 0.7 m, 1 m, and 3 m using a dichoptic rotating ring system with red-blue anaglyph glasses. Presbyopia was classified as low (LP, ADD < 1.5D) or high (HP, ADD ≥ 1.5D). Tests included dynamic sharpness discrimination, binocular integration, and stereopsis. To account for potential confounders, generalized linear models (GLM) were applied with sex, eye laterality, age, ADD, spherical equivalent (SE), and group as covariates, allowing comparison of visual function outcomes across different viewing distances between NP and ADD-stratified presbyopic groups. **Results:** There was no statistically significant difference in the passing rates of dynamic sharpness discrimination test between the presbyopic and NP groups (all *p* > 0.05). At 0.4 m, 0.7 m, and 1 m, the presbyopic group showed significantly lower passing rates in the binocular integration test compared with the NP group (all *p* < 0.05), while no significant difference was observed at 3 m (*p* = 0.051). Furthermore, the passing rates for binocular integration test at all distances were significantly lower in the HP group than those in both the NP and LP groups (all *p* < 0.05). GLM analysis indicated that both SE and age were potential confounders in the comparison of binocular integration between presbyopic and NP groups (both *p* < 0.05). There were no significant differences in the passing rates of binocular dynamic stereopsis test at any distance between the NP and presbyopic groups, or between the LP and HP groups (all *p* > 0.05). **Conclusions:** This novel dynamic testing method revealed ADD-dependent impairment of binocular integration at near-to-intermediate distances in patients with presbyopia.

## 1. Background

Presbyopia, characterized by difficulty in near vision, is an age-related decline in accommodative function, pathologically attributable primarily to reduced ciliary muscle function or decreased lens elasticity, resulting in diminished accommodative ability [1]. Presbyopia exhibits significantly increased prevalence and severity with age, reaching up to 85% among individuals aged over 40 years old [2]. Additional diopter (ADD) refers to the extra refractive power required for near vision compared with distance correction, which is a common parameter to quantify the severity of presbyopia. The binocular visual system is pivotal in overall visual function. Binocular fusion may be affected predominantly by the demands for accommodation and convergence at different viewing distances; specifically, near tasks require stronger accommodation, whereas the accommodative demand decreases with increasing distance [3]. By integrating images from both eyes in the cortex, binocular fusion can contribute to the construction of unified stereopsis, depth perception, and spatial localization [4]. However, in presbyopic individuals, reduced accommodative ability during near tasks may trigger convergence insufficiency, impaired stereopsis, unstable fusion, and decreased visual comfort [5], ultimately compromising daily work efficiency [6,7].

In recent years, great concern has been attached to the investigation on the relationship between presbyopia and binocular visual function. Existing evidence indicates that reduced stereoacuity in presbyopic patients is closely associated with aging and interocular differences in corrected visual acuity, suggesting that imbalance in binocular visual input may be an important contributor to presbyopia-related stereopsis impairment [8]. Newly diagnosed presbyopic patients often exhibit recession of the near point of convergence and reduced accommodative ability, and increased ADD values are closely correlated with aging and impaired binocular coordination, indicating that accommodative–vergence imbalance may occur even in the early stage of presbyopia [9]. In addition, reduced monocular contrast sensitivity, impaired stereopsis, and altered homeostasis of binocular suppression in older adults suggest that presbyopia-related binocular visual dysfunction may not be solely attributable to reduced ocular accommodation, but may also involve central visual integration and compensatory mechanisms [10]. Therefore, investigating the effects of presbyopia on binocular visual function may help identify the interplay among accommodative decline, binocular input imbalance, and central integration deficits, thereby informing early screening, comprehensive assessment, and individualized correction of presbyopia [5,8,11].

However, existing research primarily focuses on visual function changes at a single viewing distance, with limited systematic evaluations of binocular fusion across multiple distance conditions. With the use of a single testing tool or a target only one variable (such as accommodative amplitude), most studies failed to capture the dynamic nature of binocular fusion. Furthermore, there is a scarcity in the research examining individuals with varying ADD values under multiple distance conditions, which inhibits the development of precise visual intervention strategies and hinders the optimization of personalized correction plans. The red–blue dichoptic visual function test separates interocular visual inputs using red–blue filter glasses, allowing evaluation of monocular visual discrimination, binocular integration, interocular suppression, and stereopsis based on binocular recognition, fusion, suppression, and depth processing [12]. When combined with a multi-distance design, it further characterizes binocular visual changes under varying accommodative demands, providing a more comprehensive assessment of visual burden in real-world visual tasks [13].

In view of the above, the present case-control study was performed by employing a novel binocular visual function testing method based on a red-blue anaglyph dissociation system, using non-presbyopic (NP) individuals as controls. This study analyzed the perceptual process of dynamic rotating ring images to objectively assess sharpness discrimination, binocular integration, and binocular stereopsis in both presbyopic and NP individuals at various distances, and further investigated the impact of presbyopia severity on these visual functions.

## 2. Methods

### 2.1. Participant Inclusion Criteria

This case-control study, conducted in May 2024, enrolled 54 presbyopic and 77 NP participants at the Eye & ENT Hospital of Fudan University. All participants underwent routine ophthalmic examinations, including slit-lamp microscopy, automated and subjective refraction, best-corrected distance visual acuity (CDVA), and fundus examination.

The inclusion criteria for the presbyopic group were age ≥40 years and ADD > 0 D; while the inclusion criterion for the NP group was ADD = 0 D. The exclusion criteria for both groups were history of ocular surgery, progressive corneal disorders, cataracts, glaucoma, uveitis, and diabetic retinopathy, and history of cognitive or mental disorder. In accordance with the principles outlined in the Declaration of Helsinki, the present study was conducted with official approval given by the Ethics Committee of the Eye & ENT Hospital of Fudan University (ethics code: 2022104, date of approval 18 August 2022). All participants provided written informed consent.

### 2.2. ADD Measurement

ADD was assessed using a standard subjective refraction procedure. Binocular ADD was measured at a distance of 40 cm using the Bankoku near vision chart (Handaya Co., Ltd., Tokyo, Japan) for all patients after completing the distance vision correction. Starting from the distance prescription, convex lenses were incrementally added in steps of 0.25 D until the achievement of the optimal corrected near visual acuity and the feeling of subjective comfort with minimal accommodative effort in the participants. The final ADD value was confirmed under binocular viewing conditions. All measurements were conducted in a dark room. After ADD measurement, all participants underwent dynamic binocular visual function testing using the red–blue dichoptic system, with assessments performed independently by two technicians who were masked to each other’s results.

### 2.3. Dynamic Visual Function Measurements

**Figure 1:**
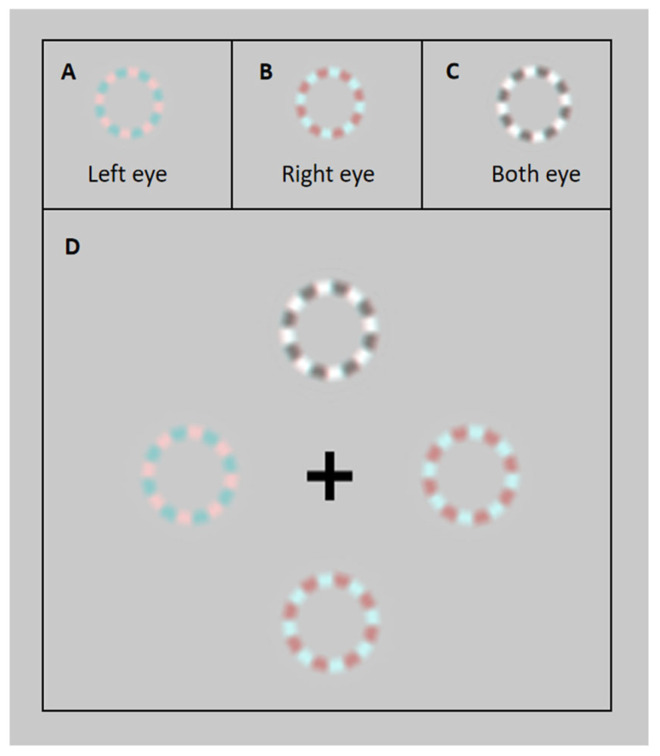
Diagram of the binocular dynamic visual function testing procedure.

In this study, dynamic visual function testing was performed using a Nubia Pad 3D (Guangdong Shiming Technology Development Co., Ltd., Guangzhou, China). The testing software was Visual Function Examination and Therapy Software (Version: SMKJ-SGN-2023 V1). Dynamic rotating ring stimuli were presented on an 11-inch tablet (resolution: 2880 × 1800 pixels; refresh rate: 60 Hz). The ring had a diameter of 10 mm and was composed of 16 equally wide segments. By wearing red-blue anaglyph glasses, subjects were instructed to view image A and image B through the left and right eyes, respectively. A composite image C was visible to both eyes, as shown in test interface D. Subjects were asked to complete this test in a standing position, maintaining eye level with the center of the screen, at distances of 0.4 m, 0.7 m, 1 m, and 3 m, respectively (Figure 1). All visual function measurements were conducted with distance correction via spectacles.

In this study, the dynamic rotating-ring task was operationally defined as a behavioral measure of dynamic binocular integration under red–blue dichoptic conditions. Correct perception of image rotation indicated effective integration of sequential stimuli presented separately to the two eyes, whereas incorrect or absent direction judgments suggested impaired integration of the dichoptic sequence. Binary outcomes are simple to administer, clinically interpretable, and suitable for preliminary screening, and may comprehensively reflect dynamic behavioral performance shaped by binocular integration, motion perception, contrast sensitivity, attentional processing, and response strategy.

### 2.4. Dynamic Sharpness Discrimination Test

Subjects were instructed to view the ring at the top on the test interface (Graph D in Figure 1) using the right eye, left eye, and both eyes separately. The ring rotated either clockwise or counterclockwise at a speed of 25 revolutions per minute (RPM). After that, each subject was asked to identify the direction of the rotation. A correct response was recorded as a pass, while an incorrect response was identified as a failure for the test.

### 2.5. Binocular Integration Test

Subjects were asked to view the ring at the bottom of the test interface (Graph D in Figure 1), which was composed of alternating presentations of Graph A and Graph B. The program displayed a sequence of “0° Graph A → 22.5° rotated Graph B → 45° rotated Graph A” at 150-millisecond intervals. This sequence, leveraging the principle of visual persistence, allowed subjects with normal binocular integration to perceive a continuously rotating ring moving clockwise at 25 RPM. However, the visual sequence would be “0° Graph A → blank → 45° (i.e., −15°) rotated Graph A” in the presence of monocular suppression (e.g., when a subject could not perceive Graph B). According to the same principle of visual persistence, such a subject would subjectively perceive a counterclockwise rotating ring at a speed of 12.5 RPM.

### 2.6. Binocular Dynamic Stereopsis Test

The stimulus comprised an 8° × 8° dynamic random-dot pattern with a mean luminance of 36 cd/m^2^ on a yellow background of 44 cd/m^2^. A central random-dot E optotype subtending 6° × 6° varied in disparity from relative zero to 800 arcsec and moved horizontally in a first-order manner. Motion speed was tested at five levels: 50, 100, 200, 400, and 600 arcsec/ms, with a 1.2-s period. Random-dot density was constant, and peripheral dots remained at relative zero disparity. Participants wore dichoptic glasses and began testing at the lowest speed. They reported the orientation of the E optotype opening via the interface. Passing required 100% accuracy at each level, with testing progressing from low to high motion speed.

### 2.7. Statistical Analysis

Statistical analyses and plotting were conducted using SPSS version 25.0 (IBM SPSS^®^ Statistics, Chicago, IL, USA) and GraphPad Prism version 8 (GraphPad Software, Ltd., San Diego, CA, USA). Continuous variables were described using mean ± standard deviation (SD). The presbyopic participants were stratified according to ADD into a low-presbyopia group (LP, ADD < 1.5 D) and a high-presbyopia group (HP, ADD ≥ 1.5 D). Baseline characteristics, including sex, age, refractive errors, CDVA, and ADD, were first assessed for homogeneity of variance. Variables satisfying the homogeneity assumption were compared using standard binary logistic regression, whereas variables violating this assumption were analyzed using Welch’s *t*-test. Generalized linear models (GLMs), including sex, eye, age, ADD, spherical equivalent (SE), and group as potential covariates, were applied to compare binocular dynamic sharpness discrimination, binocular integration, and dynamic stereopsis across viewing distances among the NP group and presbyopic subgroups, while adjusting for potential confounding factors and inter-factor effects, including the inclusion of both eyes from the same participant. In addition, the associations of ADD with binocular visual function were analyzed. Statistical significance was defined as *p* < 0.05.

## 3. Results

### 3.1. Baseline Characteristics of the Enrolled Participants

**Figure 2:**
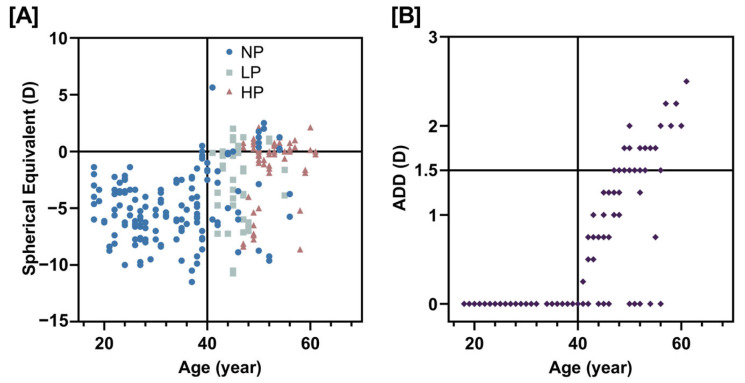
Distributions of SE (**A**) and ADD values (**B**) of subjects by age (ADD, additional diopter; NP, non-presbyopia; LP, low presbyopia; HP, high presbyopia).

Baseline demographic and clinical data of the NP and presbyopic groups are presented in Table 1. The presbyopic group showed significantly higher age and ADD values than the NP group (both *p* < 0.001). The absolute values of sphere, cylinder, and SE were all lower in the presbyopic group compared to the NP group (all *p* < 0.001). Among the presbyopic participants, the LP group included 23 patients and the HP group included 31 patients; age was significantly lower in the LP group than in the HP group (*p* < 0.05). Sex distribution did not differ significantly among groups (*p* = 0.301), and sex was not a significant influencing factor for any dynamic visual function parameter (all *p* > 0.05). The distributions of SE and ADD by age are shown in Figure 2.

### 3.2. Dynamic Sharpness Discrimination

Table 2 summarizes the results of monocular and binocular dynamic sharpness discrimination tests for all participants. There were no statistically significant differences in the passing rates of dynamic sharpness discrimination tests between the presbyopic group and the NP group (all *p* > 0.05). The passing rates of monocular dynamic sharpness discrimination tests at various distances in the HP group were slightly lower than those in the NP group and the LP group, yet without statistically significant differences (all *p* > 0.05). 

### 3.3. Binocular Integration

**Figure 3:**
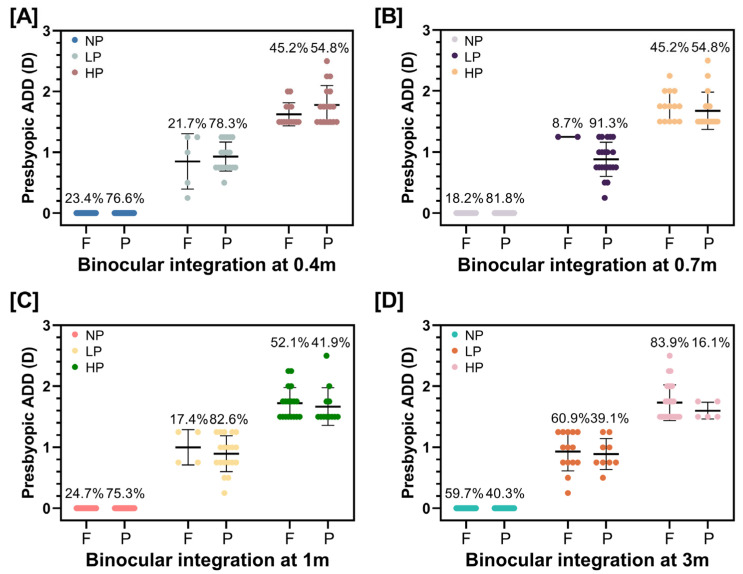
Passing rates of binocular integration tests at 0.4 m (**A**), 0.7 m (**B**), 1 m (**C**), and 3 m (**D**) in NP, LP, and HP groups (NP, non-presbyopia; LP, low presbyopia; HP, high presbyopia; ADD, additional diopter).

**Figure 4:**
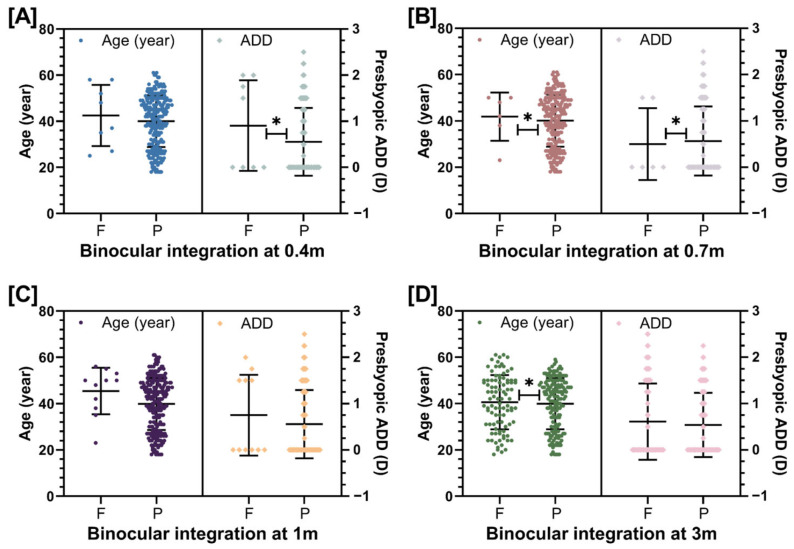
Age and ADD distribution of subjects who passed and failed the binocular integration tests at 0.4 m (**A**), 0.7 m (**B**), 1 m (**C**), and 3 m (**D**) (P, participants who passed the test; F, participants who failed the test; ADD, additional diopter). * There was a significant difference in age between F and P groups (*p* < 0.05).

As presented in Table 3 and Figure 3, GLM revealed that the presbyopic group showed significantly lower passing rates in binocular integration tests at 0.4 m, 0.7 m, and 1 m compared to the NP group (all *p* < 0.05); whereas no significant difference was found at 3 m (*p* = 0.051), with SE being identified as a significant confounder (*p* = 0.017). Moreover, passing rates at all tested distances were significantly lower in the HP subgroup than those in the NP and LP groups (all *p* < 0.05). Figure 4 displays the distributions of age and ADD values for subjects who passed or failed the binocular integration tests at different distances. Participants who passed the tests at 0.7 m and 3 m were significantly younger than those who failed, and GLM analysis identified age as a confounding factor for both test results (both *p* < 0.05).

### 3.4. Dynamic Stereopsis

The passing rates of dynamic binocular stereopsis tests are presented in Table 4. There were no significant differences in the passing rates across all distances between the presbyopic group and the NP group, as well as between HP and LP subgroups (all *p* > 0.05). 

## 4. Discussion

The binocular visual function and the impact of ADD values at different distances hold important clinical implications for presbyopic patients; however, current research and assessment methods for binocular integration in presbyopes remain limited and simplistic. Using a novel red-blue anaglyph-based dynamic testing system, this study exploratorily assessed binocular dynamic visual functions in presbyopic patients with varying ADD values across distances and aimed to characterize the visual behavioral patterns and related influencing factors in patients with presbyopia during dynamic dichoptic tasks. The main finding of this study was that ADD values significantly affected binocular integration at near (0.4 m, 0.7 m) and intermediate (1 m) distances; at the far distance (3 m), reduced binocular integration was observed only in HP patients. Overall, dynamic sharpness discrimination and dynamic stereopsis were not significantly reduced across viewing distances in patients with presbyopia, whereas binocular integration was markedly impaired with ADD- and distance-dependent patterns. Clinically, multi-distance dynamic binocular integration assessment may complement conventional near visual acuity and ADD evaluation in identifying potential binocular coordination deficits in presbyopia.

Sharpness discrimination refers to the ability to distinguish differences in visual stimulus sharpness [14] and has been used in perceptual training for presbyopia [15]. In our study, there were no statistically significant differences in the passing rates of dynamic sharpness discrimination tests at various distances between the presbyopic and NP groups (all *p* > 0.05), suggesting visual discrimination ability may remain relatively preserved in patients with presbyopia under the dynamic testing conditions. Liza et al. [16] found that alternating-distance sharpness discrimination training did not improve visual acuity more than fixed-distance training, suggesting that its effect may mainly reflect visual cortical perceptual learning rather than enhanced accommodation or ocular muscle function [17]. Meanwhile, Castro et al. [18] further showed that lower-ADD presbyopic patients better tolerated anisocoria-induced contrast sensitivity loss and binocular visual decline while preserving binocular summation, indicating retained compensatory visual discrimination in early presbyopia. Moreover, HP participants showed slightly lower passing rates of monocular dynamic sharpness discrimination tests at all distances compared to the NP and LP participants, despite the presence of no statistically significant differences, further supporting the hypothesis that patients with presbyopia may maintain dynamic visual discrimination ability within a certain range through visual perceptual compensation. A possible explanation is that adult visual neural plasticity enables contrast compensation via contrast gain control when optical image quality is degraded. This mechanism may partially offset defocus-induced optical blur in presbyopia, thereby preserving visual discrimination and perceptual quality [19,20]. Therefore, this study suggests that dynamic sharpness discrimination may not be the most sensitive indicator of functional impairment in patients with early-stage or low-ADD presbyopia.

Binocular vision relies on dynamic accommodation–vergence balance mediated by the accommodative convergence/accommodation (AC/A) ratio. Abnormal AC/A may cause asthenopia, diplopia, and stereopsis impairment [21]. This study revealed significantly lower binocular integration passing rates in the prebyopic group at 0.4 m, 0.7 m, and 1 m compared to the NP group, whereas the difference at 3 m was not significant (*p* = 0.051). This distance-dependent result suggests that dynamic dichoptic integration at near-to-intermediate distances, which entail higher accommodative and vergence demands, may more readily reveal binocular integration differences in presbyopic individuals. This aligns with the findings of Granger-Donetti et al. [22], which reported binocular cooperation dysfunction in most presbyopes, partly due to reduced convergence velocity. At the far distance of 3 m, however, no significant difference was observed in the passing rate of binocular integration tests between the presbyopic group and NP group, suggesting a dominant role of vergence under low accommodative and vergence demand at far distances [23]. This aligns with Jain et al. [24], who found that near correction in presbyopia improved distance accommodative flexibility without significantly affecting near phoria or accommodative flexibility, supporting a distance-dependent effect of ADD on binocular integration. Rozanova et al. [25] also noted that proximal fusion limits change more markedly than distal fusion limits in presbyopes. Therefore, these findings suggest that near-to-intermediate visual tasks may place greater demands on binocular integration in patients with presbyopia.

Additionally, the passing rates of binocular integration tests at all distances were significantly lower in HP group than those in the NP group and LP group (all *p* < 0.05). Higher ADD levels were associated with poorer performance in the dynamic dichoptic integration task, indicating that ADD may help identify presbyopic patients with impaired task performance. As ADD increases, the impact of presbyopia on binocular vision may extend from simple near accommodative insufficiency to a more generalized decline in accommodative–vergence coordination across multiple viewing distances. Alvarez et al. [26] reported individual variability in adaptation to progressive addition lenses (PAL), with early presbyopes showing greater phoria adaptation, enhanced convergence, and faster adaptation. In the present study, low-ADD presbyopes may retain relatively preserved fusional reserves and binocular integration because accommodation is not yet markedly impaired. In contrast, higher-ADD patients showed reduced integration across multiple distances, possibly related to greater near visual demand, binocular coordination deficits, or age-related visual processing changes. The increased accommodative burden may further disrupt accommodation–vergence balance, impair binocular integration, and contribute to visual fatigue, reading difficulty, and reduced stereopsis [27], with effects dependent on ADD and viewing distance. Therefore, these findings suggest that ADD level may help identify presbyopic individuals with poorer performance in dynamic dichoptic integration tasks.

GLM analysis identified SE and age as confounders of binocular integration performance (both *p* < 0.05). Higher myopia and stronger spectacle correction may alter spectacle magnification and retinal image size, inducing aniseikonia, peripheral distortion, or prismatic effects, thereby affecting binocular balance, fusion stability, and stereoscopic processing [28]. Higher myopia may reduce contrast sensitivity, and contrast-related retinal input degradation may further impair target recognition, motion-direction discrimination, and binocular integration during dynamic stimulation [29]. Meanwhile, Participants who passed the binocular integration test at 0.7 m and 3 m were significantly younger than those who failed (both *p* < 0.05), suggesting age as an important determinant of dynamic binocular integration. Aging-related declines in contrast sensitivity, stereoacuity, interocular suppression balance, and binocular processing may reflect reduced cortical visual integration efficiency [10]. Similarly, Sepulveda et al. reported that age-related declines in motion perception and temporal processing may affect judgments of dynamic rotating stimuli [30]. Future studies should include age- and SE-matched controls and objective measures of accommodation, vergence, motion perception, and reaction time to clarify the relative contributions of aging, refractive status, and presbyopia.

Stereopsis is the perception of depth formed by integrating slightly different images from both eyes, depending on binocular disparity, binocular integration, and neural processing of disparity in the brain [25,31,32]. Ochi et al. [33] reported that bilateral low-Add multifocal intraocular lenses (IOLs) achieved better intermediate and near stereopsis than monofocal IOLs, suggesting that ADD correction may improve stereopsis. This study found that dynamic stereopsis pass rates did not differ between NP and presbyopic groups or among ADD subgroups at any distance (all *p* > 0.05), suggesting that impaired binocular integration does not necessarily compromise dynamic stereopsis, which may involve distinct compensatory mechanisms. One possible reason is that previous studies used static stereopsis tests, such as Titmus, TNO, and Randot [34], which may not fully capture dynamic visual integration. Watanabe et al. [35] found that 3 of 7 strabismus patients without static stereopsis could perceive depth motion, suggesting that dynamic stereopsis testing better reflects depth perception in real dynamic environments and may be more sensitive to certain visual dysfunctions than static tests. Compared with static fixation, dynamic fixation may enhance stereopsis by providing time-varying disparity and interocular velocity differences, thereby enriching dynamic depth cues [32]. These dynamic depth cues may partially compensate for age-related declines in fusional ability, allowing patients with presbyopia to preserve dynamic depth perception despite impaired binocular integration. These findings suggest that presbyopic visual assessment should distinguish static stereopsis, dynamic stereopsis, and binocular integration, rather than using any single binocular measure to represent overall binocular function. Given the low dynamic stereopsis pass rate, future studies should validate these results with conventional tests, such as Titmus, TNO, and Randot, and systematically compare static and dynamic stereopsis across populations.

This study still has several limitations. First, the red–blue dichoptic dynamic rotating-ring system is a novel paradigm requiring further validation of repeatability, sensitivity, specificity, and test–retest reliability. Second, binary pass/fail outcomes, although clinically simple, may reduce sensitivity, obscure subtle differences, and limit continuous quantification of dynamic binocular dysfunction. Third, because accommodative amplitude, vergence function, and visual fatigue were not directly assessed, explanations of ADD-related binocular integration mechanisms and potential benefits of visual training remain hypothetical and require objective validation.

## 5. Conclusions

This novel dynamic testing method revealed impaired binocular integration in patients with presbyopia, with impairment varying according to ADD level and viewing distance. The findings of this study provide a novel approach for assessing dynamic binocular visual function, and may facilitate early identification of declining binocular integration in presbyopic individuals.

## Figures and Tables

**Table 1:** Demographics of enrolled participants.

Characteristic	Mean ± SD	Presbyopia Group			NP Group	*p* ^1^
		LP Group	HP Group	All		
Age (years)	40.14 ± 11.26	45.70 ± 3.13 *	52.23 ± 3.84 *	49.44 ± 4.80	33.53 ± 9.75	**<0.001**
Gender (male/female)	41/90	5/18	10/21	15/39	26/51	0.301
Refraction sphere (D)	−3.21 ± 3.29	−2.37 ± 3.32	−1.31 ± 2.95	−1.76 ± 3.14	−4.23 ± 3	**<0.001**
Refraction cylinder (D)	−0.76 ± 0.83	−0.60 ± 0.76	−0.50 ± 0.47	−0.54 ± 0.61	−0.91 ± 0.93	**<0.001**
SE (D)	−3.6 ± 3.42	−2.67 ± 3.44	−1.56 ± 3.02	−2.03 ± 3.24	−4.69 ± 3.12	**<0.001**
CDVA (LogMAR)	0 ± 0.01	0 ± 0.01	0.01 ± 0.02	0 ± 0.02	0 ± 0.01	0.083
ADD (D)	0.56 ± 0.74	0.91 ± 0.28 *	1.71 ± 0.27 *	1.37 ± 0.48	0 ± 0	**<0.001**

* LP group vs. HP group; *p* < 0.05; ^1^ Presbyopia group vs. NP group; Abbreviations: LP, low hyperopic; HP, high hyperopic; NP, non-presbyopic; SE, spherical equivalent; CDVA, corrected-distant visual acuity; ADD, additional diopter. Values with statistical significance between groups are shown in bold.

**Table 2:** Passing rates of sharpness discrimination tests at four distances of presbyopic and NP groups.

Distances	NP Group	Presbyopic Group			*p* ^a^
		LP Group	HP Group	All	
0.4 m	150.0 (97.4%)	46.0 (100.0%)	58.0 (93.5%)	104.0 (96.3%)	0.128
0.7 m	150.0 (97.4%)	46.0 (100.0%)	60.0 (96.8%)	106.0 (98.1%)	0.609
1 m	148.0 (96.1%)	46.0 (100.0%)	57.0 (91.9%)	103.0 (95.4%)	0.285
3 m	96.0 (62.3%)	36.0 (78.3%)	35.0 (56.5%)	71.0 (65.7%)	0.250
0.4 m (Bi)	77.0 (100.0%)	23.0 (100.0%)	30.0 (96.8%)	53.0 (98.1%)	0.998
0.7 m (Bi)	75.0 (97.4%)	23.0 (100.0%)	31.0 (100.0%)	54.0 (100.0%)	1.000
1 m (Bi)	77.0 (100.0%)	23.0 (100.0%)	31.0 (100.0%)	54.0 (100.0%)	1.000
3 m (Bi)	69.0 (89.6%)	23.0 (100.0%)	29.0 (93.5%)	52.0 (96.3%)	0.112

Abbreviations: Bi, Binocular; NP, non-presbyopic; LP, low presbyopic; HP, high presbyopic. ^a^ presbyopic group versus NP group.

**Table 3:** Passing rates of binocular integration tests at four distances of presbyopic and NP groups.

Distances	NP Group	Presbyopic Group			*p* ^a^
		LP Group	HP Group	All	
0.4 m	59.0 (76.6%)	18.0 (78.3%)	**17.0 (54.8%)** **^b,c^**	35.0 (64.8%)	**0.027**
0.7 m	63.0 (81.8%)	21.0 (91.3%)	**17.0 (54.8%)** **^b,c^**	38.0 (70.4%)	**0.002**
1 m	58.0 (75.3%)	19.0 (82.6%)	**13.0 (41.9%)** **^b,c^**	32.0 (59.3%)	**0.001**
3 m	31.0 (40.3%)	9.0 (39.1%)	**5.0 (16.1%)** **^b,c^**	14.0 (25.9%)	0.051

Abbreviations: NP, non-presbyopic; LP, low presbyopic; HP, high presbyopic. ^a^: presbyopic group versus NP group; ^b^: presbyopic group versus NP group, *p* < 0.05; ^c^: presbyopic group versus LP group, *p* < 0.05. Values with statistical significance between groups are shown in bold.

**Table 4:** Passing rates of dynamic stereopsis tests at four distances of presbyopic and NP groups.

Distances	NP Group	Presbyopic Group			*p* ^a^
		LP Group	HP Group	All	
0.4 m	47.0 (61.0%)	13.0 (56.5%)	16.0 (51.6%)	29.0 (53.7%)	0.805
0.7 m	46.0 (59.7%)	14.0 (60.9%)	15.0 (48.4%)	29.0 (53.7%)	0.923
1 m	41.0 (53.2%)	13.0 (56.5%)	16.0 (51.6%)	29.0 (53.7%)	0.797
3 m	33.0 (42.9%)	11.0 (47.8%)	10.0 (32.3%)	21.0 (38.9%)	0.622

Abbreviations: NP, non-presbyopic; LP, low presbyopic; HP, high presbyopic. ^a^: presbyopic group versus NP group.

## Data Availability

Data underlying the results presented in this paper are not publicly available at this time but may be obtained from the authors upon reasonable request.
